# Long-term changes in winter abundance of the barbastelle *Barbastella barbastellus* in Poland and the climate change – Are current monitoring schemes still reliable for cryophilic bat species?

**DOI:** 10.1371/journal.pone.0227912

**Published:** 2020-02-18

**Authors:** Iwona Gottfried, Tomasz Gottfried, Grzegorz Lesiński, Grzegorz Hebda, Maurycy Ignaczak, Grzegorz Wojtaszyn, Mirosław Jurczyszyn, Maciej Fuszara, Elżbieta Fuszara, Witold Grzywiński, Grzegorz Błachowski, Janusz Hejduk, Radosław Jaros, Marek Kowalski

**Affiliations:** 1 Department of Behavioural Ecology, University of Wroclaw, Wroclaw, Poland; 2 Polish Society of Wildlife Friends “pro Natura”, Wroclaw, Poland; 3 Institute of Animal Sciences, Warsaw University of Life Sciences, Warsaw, Poland; 4 Institute of Biology, University of Opole, Opole, Poland; 5 The Polish Society for Bat Protection, Poznan, Poland; 6 Polish Society for Nature Conservation "SALAMANDRA", Poznan, Poland; 7 Department of Systematic Zoology, Adam Mickiewicz University in Poznan, Poznan, Poland; 8 Faculty of Biological Sciences, Cardinal Stefan Wyszynski University in Warsaw, Warsaw, Poland; 9 Department of Animal Physiology, University of Warsaw, Warszawa, Poland; 10 Faculty of Forestry, Poznan University of Life Sciences, Poznan, Poland; 11 Department of Biodiversity Studies, Teacher Training and Bioeducation, University of Lodz, Lodz, Poland; 12 Wildlife Society 'STORK', Warsaw, Poland; Nanjing Forestry University, CHINA

## Abstract

Warmer winters may lead to changes in the hibernation behaviour of bats, such as the barbastelle *Barbastella barbastellus*, which prefers to hibernate at low temperatures. The species is also known for its large annual fluctuations in the number of wintering individuals, so inference about population trends should be based on long-term data. Prior to 2005, analyses indicated stable or even increasing barbastelle population in Poland. We analysed the results of 13 winter bat counts (2005–2017) of the species from 15 of the largest hibernacula, and additional site of 47 small bunkers, in Poland. The total number of wintering individuals remained stable during the study period, because the barbastelle is not a long-distance migrant, this likely reflects the national population trend. On the basis of mean winter air temperatures we divided the country into four thermal regions. Analyses of barbastelle abundance in hibernacula in the four regions revealed a 4.8% annual mean increase in numbers in the coldest region, where mean winter temperatures were below -2°C, annual mean declines of 3.3% and 3.1% in two warmer regions of western Poland, but no trend in the region of intermediate mean winter temperatures of between -1°C and -2°C. Overall, there was a significant, but weak, negative correlation between the abundance of hibernating individuals and the mean winter temperature. On the other hand, the number of individuals hibernating in small bunkers increased, even though the site was located in one of the warm regions. The results indicate a warming climate will likely reduce the use of large, well-insulated winter roosts by species that prefer colder conditions–and that this is already happening. For forest-dwelling bats, such as the barbastelle, for which monitoring schemes are primarily based on winter surveys of large hibernacula, estimations of population trends may consequently become less reliable.

## Introduction

Climatic changes, especially changes of temperature, are one of the most important factors affecting populations of organisms, directly affecting the density and distribution of species and indirectly influencing habitat occupation, microclimate of shelters, and changes in the number of prey or predators [1, 2, 3]. A warming climate can result in earlier breeding in amphibians and birds [4, 5, 6], and markedly modify the occurrence of mammal species [7, 8], with Levinsky et al. [9] estimating that up to 9% of European mammals may consequently become extinct in the 21^st^ century, and a further 32–78% may become endangered with their ranges reduced by over 30%.

Currently, the greatest temperature increases are noted in the climate of the Northern Hemisphere [10, 11, 12], particularly in the winter and spring months [13, 14, 15], with a decadal increase of 0.5–1% occurring mostly over autumn and winter in the mid to high latitudes [16]. For some mammals, such as bats, an increase of minimum and mean temperatures, especially in winter seem to have the most significant impacts [9, 17], including directly affecting their prey detection ability (species emitting high frequency echolocation pulses lose and species using lower frequencies gain prey detection volume) [8], and strongly influencing their and hibernation regime, potentially affecting individual survival (shorter hibernation may result in shortened life in bats) and reproductive success [9, 18, 19, 20, 21]. The reproductive cycle of temperate zone bats is closely linked to their pattern of hibernation. Temperate species mate in autumn and winter and spermatozoa are stored in the female reproductive tract until spring. If winters become shorter, females could arouse from hibernation early, ovulate and become pregnant [19]. Climate warming could also adversely affect reproductive success because spermatozoa, stored in females or in males (in their epididymis), may lose their viability if the bats are not provided with conditions suitable for hibernation [19]. Moreover, climate warming may alter species ranges[7, 22] and wintering strategies. As a result, long-distance migratory bat species, such as Nathusius’ pipistrelle *Pipistrellus nathusii*, which moves from colder parts of the breeding range to hibernate in areas with mean temperature above 0°C [23], may begin shortening their migration distance or even forming sedentary populations in the breeding range [20, 24], as the 0°C isotherm shifts.

Cryophilic bats that hibernate at low temperatures, such as the barbastelles *Barbastella barbastellus*, are particularly sensitive to temperature changes [7, 25]. During hibernation, barbastelles occupy structures with a temperature of -1°C to +6°C [25, 26, 27], usually only slightly exceeding 0°C, with either strong airflow or frost [28, 29, 30]. Such conditions are too extreme for many bat species [25, 26], which differ from barbastelles in their preferred conditions for hibernation [31, 32].

Research has shown a pronounced effect of ambient temperature on the total winter energy requirements of bats, including barbastelles, with a relatively narrow combination of hibernaculum temperature and winter length permitting successful hibernation [17, 33]. A shift to warmer and shorter winters [11, 13, 34], could change the barbastelles’ winter roost choice in two ways. Firstly, large and well-insulated hibernacula may not cool down enough (or perhaps may cool down too slowly) to permit successful hibernation of barbastelles [35, 36, 37]. Hibernating at warmer temperatures leads to increased energy expenditure [38, 39], and so individuals may maximize winter survival and optimize their energetic condition for spring emergence by choosing a hibernaculum with an ambient temperature near that of the minimum torpid metabolic rate [39, 40]. Unlike food-caching species, fat-storing hibernators, such as bats, generally cannot adjust their energy intake during hibernation; therefore, survival costs are potentially large if an animal expends energy too quickly or if an unusually harsh winter extends the hibernation season [39].

To mitigate the potential costs of warmer winters, cryophilic bats that hibernate at low temperatures, such as the barbastelle, can modify their behaviour by occupying shallower, smaller roosts, e.g. free-standing bunkers, which offer lower temperatures than larger roosts situated deeper underground [28, 29, 41]. In a study performed in a group of small bunkers in the eastern part of Poland, changes in mean outside temperature explained 91% changes of air temperature inside and mean number of barbastelles present corresponded with mean air temperature. The bats appeared in the bunkers when mean monthly temperature dropped below 0°C, in late November or during December [28]. In such conditions, barbastelles, can minimize the energy requirements of hibernation [33], and so emerge in spring with larger energy reserves that may confer a reproductive advantage [39]. Another possibility (not excluding the first one) is that due to climate warming some small, poorly insulated roosts (summer or transitional roosts) may not become too cold for barbastelles during the entire hibernation season. The species usually arrives in large hibernacula gradually, probably leaving other roost types only after the temperature inside drops below a certain point. During a study in central Poland the number of barbastelles (counted every two weeks) increased until the end of January (E. Fuszara, M. Fuszara, unpublished). Some individuals were present in tree crevice in January (I. Gottfried, T. Gottfried, unpublished) and barbastelle was found in an outside firewood stack in January (I. Gottfried, T. Gottfried, unpublished). This way or the other, milder winters could cause the barbastelles’ absence in large hibernacula.

Understanding the effect of climate change on organisms is important for protecting nature and managing natural resources at the global scale [7, 20, 42]. This is particularly relevant to the barbastelle, a species occurring almost exactly in Europe and, according to IUCN, declining since 2012 [43]. Admittedly, a report on trends in European bat populations based on a prototype biodiversity indicator indicates a moderate upward trend in the barbastelle. This is, however, based on monitoring data from just six countries and thus far from covering the entire species range. The authors state that “Due to the preliminary nature of this prototype indicator, the conclusion that bats have increased at hibernation sites should be considered with caution (…) Since combining species trends for an indicator has the potential to mask contrasting trends at species or country level, national surveillance schemes should work towards wide publication of species trends, so as to spotlight such disparities” [44], which is exactly what our paper does. Numerous studies show that barbastelle populations in Central Europe are stable [45, 46, 47, 48, 49, 50] while those in the west of the continent are decreasing [51, 52, 53, 54, 55]. In Eastern Europe the species is rather poorly studied and no population trends are known from that part of the continent. More data is available only from Ukraine, where the numbers of barbastelles seem stable or even increasing [56].

The aim of the current study was to analyse 13 years of changes in the numbers of barbastelles wintering in large, regularly surveyed hibernacula together with winter temperature data in order to identify trends and look for possible effects of weather conditions on the presence of barbastelles in the roosts under study. To determine the effect of climate change on the barbastelle more comprehensively, we additionally analysed results of bat counts in a group of smaller roosts (concrete bunkers). Assuming that climate warming makes shallower and more rapidly cooling structures [28] more appropriate for hibernating bats, we expected the number of barbastelles to increase in such hibernacula. We also discuss if monitoring barbastelle abundance based exclusively on data from large hibernacula adequately represents real population status, in view of possible change in the selection of wintering sites in relation to climate change.

## Methods

**Fig 1:**
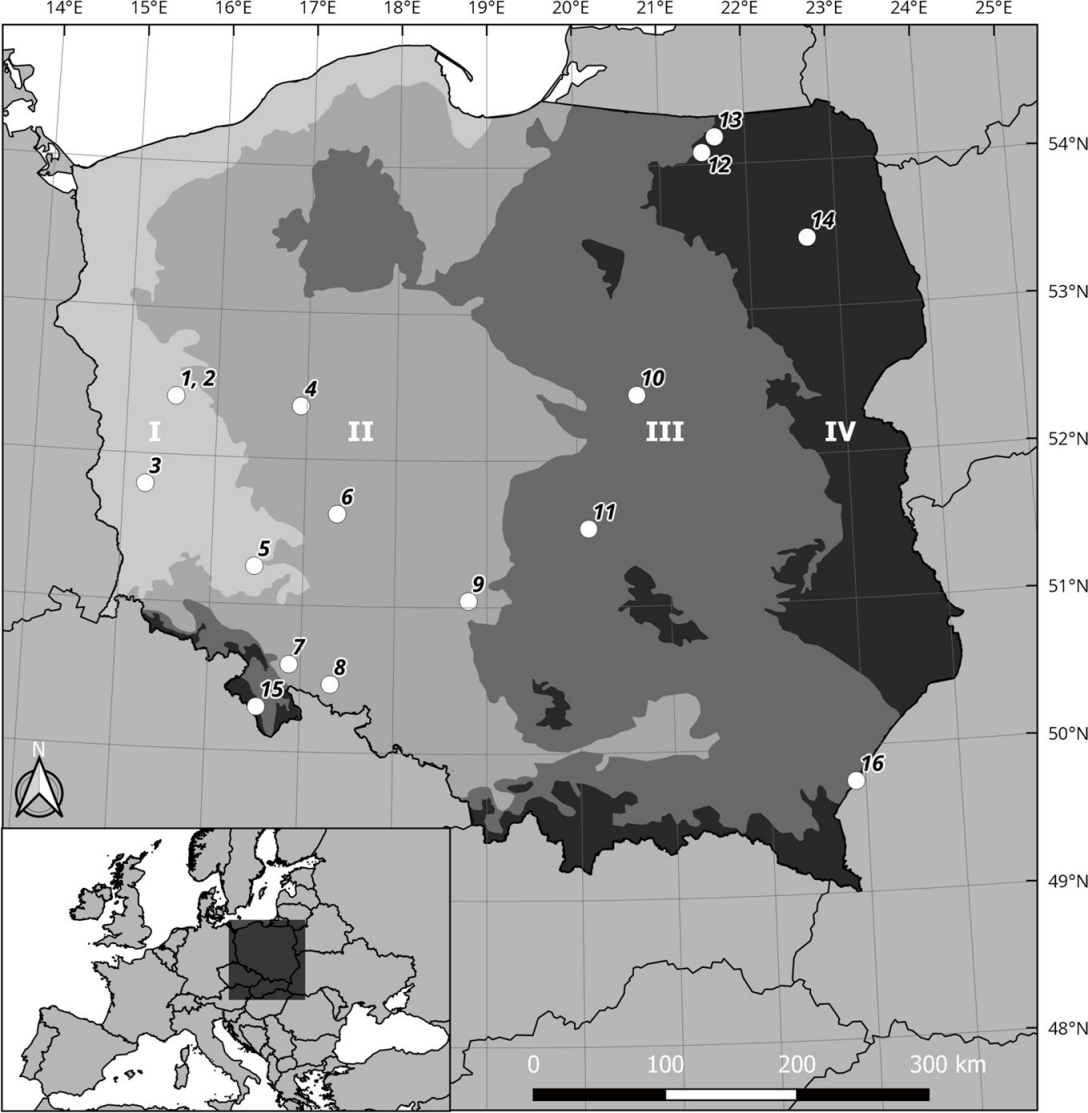
Distribution of the hibernation sites of *Barbastella barbastellus* under study in four thermal regions. The map of thermal regions was created on the basis of the map of mean winter temperatures by the Institute of Meteorology and Water Management IMGW for years 1971–2000, available at http://klimat.pogodynka.pl/pl/climate-maps/?. Thermal Regions: I (> 0°C), II (0°C to -1°C), III (-1°C to -2°C), IV (< -2°C). The numbering of the hibernation sites (1–16) corresponds to that in Table 2.

We used data from winter censuses of barbastelles performed in 2005–2017 in large hibernacula across Poland. For each hibernaculum, one count was made in a given season, between 1^st^ January and 15^th^ February (the period of the national bat census in Poland). Data were derived from regularly visited hibernacula of barbastelles where the maximum number of bats typically exceeded 100 individuals (Fig 1). In some cases (the forts in Poznań, the forts in Nysa, the forts in Modlin, railway bunkers in Konewka and Jeleń) several structures situated close to each other were treated as a single site. Consequently, we performed analyses for 15 sites, hereafter referred to as ‘large’ wintering sites or hibernacula. These sites are the largest and most important barbastelle hibernacula in Poland [29, 57, 58]–tunnels, large cellars, disused mines and fortifications, with a cubature of > 6000 m^3^. To look more closely at barbastelle numbers dynamics in different climatic conditions, we identified (on the basis of mean winter temperatures) four thermal regions in Poland. The mildest winters in the country (the warmest Region I: mean winter temperature > 0°C) occur along the northern coastal belt (Baltic Sea) and western border of the country, and the most severe winters are in eastern and north-eastern Poland, and the Sudety Mountains in the south-west (the coldest Region IV: mean winter temperature < - 2°C). Intermediate regions occur in central Poland, with mean winter temperatures of 0°C to—1°C (Region II) and—1°C to—2°C (Region III). The map of the thermal regions was created by us in QGIS software on the basis of the map of mean winter temperatures from the period 1971–2000 by the Institute of Meteorology and Water Management—National Research Institute (hereafter referred to as IMGW) [59, 60]. We then plotted the locations of all surveyed hibernacula on the thermal regions map (Fig 1).

Furthermore, results of bat counts in 47”small” (12–1000 m^3)^ roosts [41] (treated as a single site), were available from stand-alone bunkers belonging to the Międzyrzecz Fortified Region (hereafter referred to as MFR) but not physically connected to the main system of 30 km of underground corridors (i.e. the “Nietoperek” Bat Reserve). Results from the “small” sites were not included in correlation analyses of the number of barbastelles and weather conditions, nor in calculations of overall population trends in Poland, because these structures are usually small, shallow, less insulated from external temperatures. As such, the microclimate of these bunkers differs from that of the large winter roosts, being less stable than that of the major underground corridors of the MFR [28, 61] despite the proximity of both sites. The small bunkers sites are partially destroyed with different degrees of preservation and allowing extensive air circulation and temperature changes, which results in partial freezing conditions [38]. The barbastelle is typically one of the most numerous bat species in the small bunkers, numbering 1–19 individuals in single structure [41]. Therefore, we performed separate analysis for these small roosts.

### Data analyses

All analyses were conducted in R (R Core Team, 2015). Firstly, we used the *rtrim* package [62, 63] to assess the long-term changes in number of barbastelle hibernating in the large sites. This package is a reimplementation of the original TRIM (Trends and Indices for Monitoring Data) software developed to analyse monitoring data from incomplete counts using log-linear Poisson regression [64]. Analysis was performed using the ‘time effects’ model (model 3) assuming that populations vary across sites, but show the same growth everywhere and time effects are independent for each time point [64]. We employed models with a correction for over dispersion and serial correlation, and used trend estimates based on the imputed slope. In the first step, a national trend for barbastelles was computed using the full dataset with all large hibernacula. Then, the same model type was performed separately for the four thermal regions to check for differences between the warmer and colder regions of Poland.

Population trends were assigned to one of six categories depending on whether the yearly rate of change over the study period was more or less than 5%. As such, a strong increase or decrease was represented by a significant change of > 5% per year, and a moderate increase or decrease by significant changes of < 5% per year, with a stable trend for non-significant changes being < 5% and uncertain trend for non-significant changes > 5% per year. We considered a trend non-significant if confidence intervals contained 1.00.

Secondly, we performed linear regression models to determine the effect of winter thermal conditions on the number of hibernating barbastelles. The average winter temperature (for December-February) and number of frosty days (i.e. days with maximum daily temperature below 0ºC) in winter seem to have the greatest effect on the distribution of bats in temperate zones [7, 20, 21]. Mean temperatures recorded by the IMGW at weather stations closest to the hibernacula under study were acquired via the Internet from NOAA [65] and OGIMET [66]. The numbers of frost days were taken from services analyzing climate data, such as Weatheronline [67]. Both these climatic variables were strongly correlated in our dataset (r = -0.917, p < 0.001), thus we performed analyses only for mean winter temperature. Due to large differences in the number of hibernating barbastelles between the large hibernacula, the abundance at each site was standardized to an index according the following formula:

$$index\;of\;abundance=N_{ij}/N_{bj},$$

where N_ij_ = number of bats in *i*-th year at the *j*-th site, N_bj_ = number of bats in the base year at the *j*-th site. As a result, the index of abundance was the proportion of the abundance recorded in each year relative to the abundance in the first year (i.e. base year) of monitoring (2005 in our research) at the same site. Additionally, an index of abundance (as a dependent variable) was log-transformed to an approximate normal distribution for linear regression models, and also for the calculation of Pearson's correlation coefficient. These statistical analyses were used to calculate the national trend and also individual trends for the four thermal regions, using data only from the large sites. In some years, winter censuses of bats were not performed at certain sites or their parts and these cases were removed from regression models (Gierłoż 2008 and Mamerki 2008, 2014, 2015, Lubiąż 2009, 2012, Poznań 2009, 2011). Furthermore, due to the lack of counts for the baseline year (2005) at the Przemyśl site, it was impossible to calculate an index of barbastelle abundance for subsequent years. Therefore, data from this site were also excluded from regression models.

In order to check for differences in population trends between small and large structures in the same thermal region, results of censuses performed in the small bunkers of the Międzyrzecz Fortified Region and in the “Nietoperek” Bat Reserve (the main system of underground corridors of MFR) were compared. To check for a consistently increasing or decreasing number of the barbastelles hibernating in these large and small sites (as well as in other sites under study) we performed the Mann-Kendall (M-K) trend test [68], with the *EnvStats* package [69]. This non-parametric test is robust, and can cope with extreme and missing values, requiring no assumptions of a specific distribution in the data [70]. Trends were evaluated using the Z coefficient estimation; a positive and negative Z values indicated a respective upward or downward trend.

To determine statistical significance of trends, we tested a null hypothesis (H_0_) assuming no trend in the time series, and alternative hypothesis being (H_a_) that there is a trend in the time series for a given significance level. Because the detection of a trend may not be possible at α = 0.05 in the case of small sample size [71], in this study we report trends detectable at both 0.05 and 0.1 significance level.

Trends in the mean winter temperature at the national and regional level were determined using the M-K test, with the methodological assumptions described above.

### Permits

All procedures performed in studies were in accordance with ethical standards. All applicable national and European guidelines for bat monitoring were followed. We have permissions from Regional Directors for Environmental Protection for the monitoring bat hibernacula.

## Results

### Barbastelles numbers across the country and in each thermal regions

**Fig 2:**
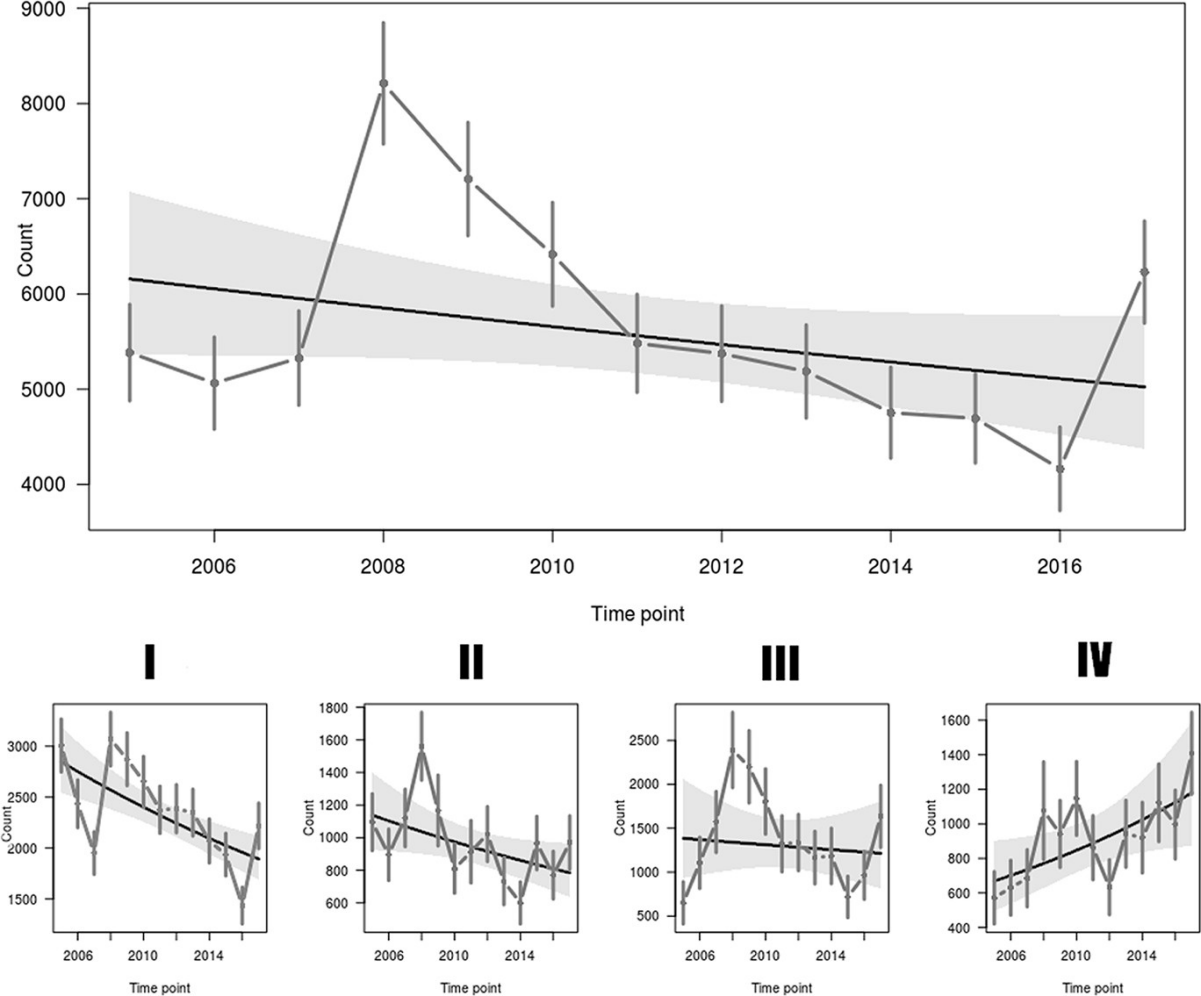
Trends in the abundance of *Barbastella barbastellus* in hibernacula in Poland. Data for large hibernation sites (upper plot) and in thermal regions I-IV (bottom plots). The overall slope and the total number of individuals per year with their 95% confidence intervals (as shaded area and vertical lines, respectively) are shown.

**Fig 3:**
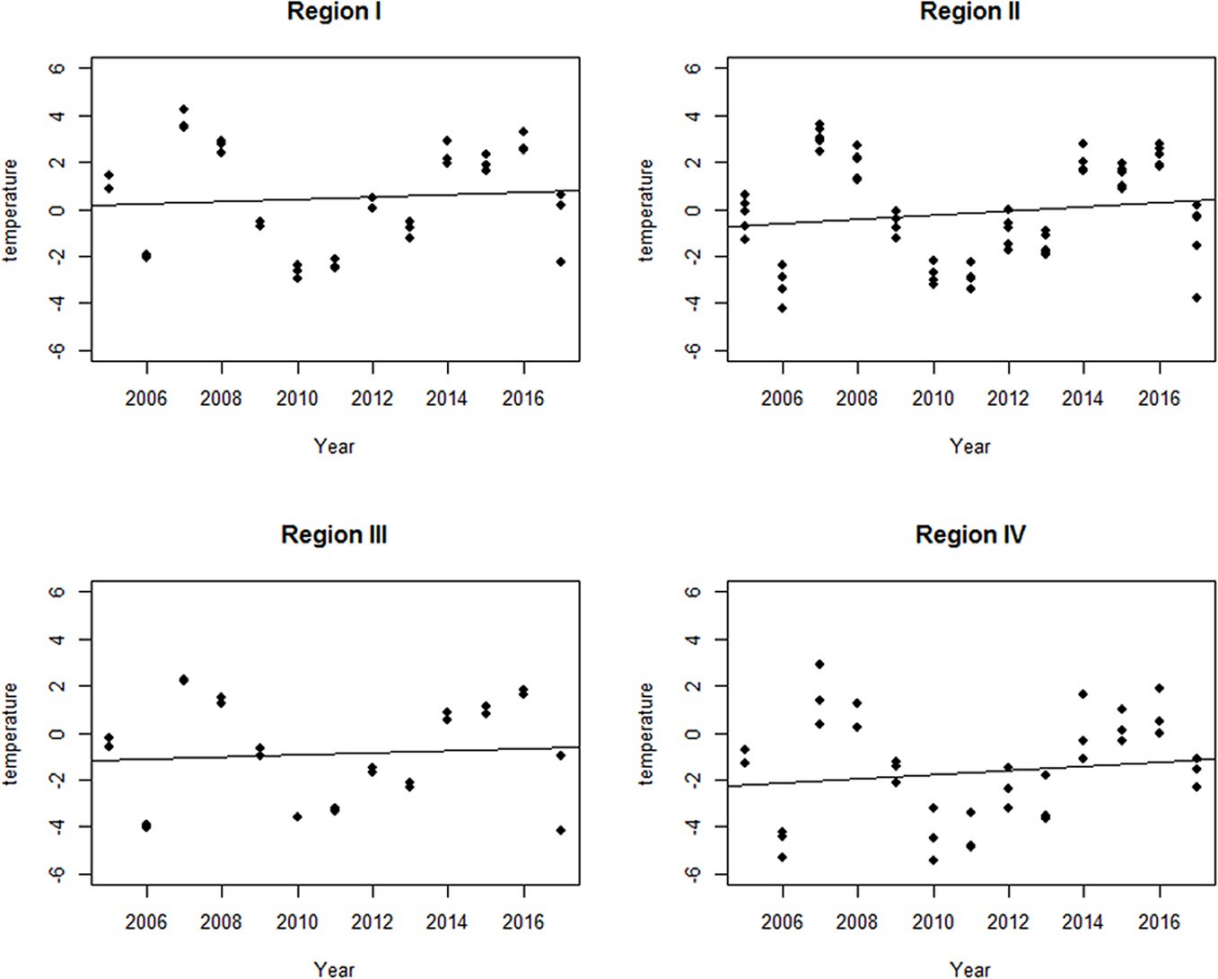
Trends in mean winter temperatures in the four thermal regions. Calculations were performed on the basis of temperatures recorded in weather stations closest to hibernacula under study.

The number of barbastelles recorded during winter bat censuses in the period 2005–2017 in the 14 largest hibernacula in Poland (excluding Przemyśl and the small bunkers around MFR; see Methods) varied between 4,164 and 7,597 individuals. The number of barbastelles decreased from 2008 (Fig 2). However, *rtrim* analysis showed an overall stable trend throughout the study period at the national level, with differences between the four thermal regions (Fig 1), indicating a moderate increase (mean 4.8% per year) in numbers in the coldest region (mean winter temperatures below -2°C) and a moderate decrease (mean 3.3% and 3.1% per year) in the warmer, western regions of the country. An indeterminate trend was apparent in the region with a mean winter temperature between -1°C and -2°C (Table 1, Fig 2, Fig 3).

**Table 1:** Trend estimates (as multiplicative slope of the regression line based upon imputed indices) and standard errors for the number of hibernating individuals of the barbastelle *Barbastella barbastellus* in Poland and in four thermal regions of the country, identified on the basis of mean winter temperature. Slope value represents an average yearly change, e.g. 0.983 imply an average decrease of 1.7% per year and 1.048 –an average increase of 4.8% per year; all slope values are statistically significant at p < 0.001.

Region	Multiplicative slope ± SE	Trend description
Whole country	0.983 ± 0.009	Stable
Region I (> 0°C)	0.967 ± 0.007	Moderate decrease (p < 0.001)
Region II (0°C to -1°C)	0.969 ± 0.013	Moderate decrease (p < 0.05)
Region III (-1°C to -2°C)	0.989 ± 0.025	Uncertain
Region IV (< -2°C)	1.048 ± 0.020	Moderate increase (p < 0.05)

Analyses showed the lack of significant trend in mean winter temperatures recorded in weather stations closest to hibernacula under study (Z = 0.305, p = 0.760) and no significant trend in individual thermal regions also (Region I: Z = 0.305, p = 0.760; Region II: Z = 0.427, p = 0.669; Region III: Z = 0.305, p = 0.760; Region IV: Z = 0.427, p = 0.669) (Fig 3). No significant differences were found for individual winter months.

Despite the lowest overall count of individuals being noted in 2016, the subsequent and final season of study (2017) found an increase of 1,969 barbastelles in the large sites, although the mean winter temperature was 1.8°C higher than in 2016 (1.1°C); in 2017 season (December 2016 –February 2017) mean temperatures were higher than in 2016 season (December 2015 –February 2016) for December (4.8°C vs 1.1°C) and February (3.2°C vs 0.9°C), but not January (-2.4°C vs 1.3°C).

The number of barbastelles in the large hibernacula showed a significant, but weak, negative correlation with the average winter temperature (r = -0.305, p < 0.001). With the linear regression model showing that this climatic factor explained 9.3% of the variance in bat abundance. Analyses for individual thermal regions showed a similar effect of mean winter temperature on the number of hibernating barbastelles in the warmer western regions (Region I: r = -0.393, r^2^ = 0.155, p = 0.016; Region II: r = -0.309, r^2^ = 0.096, p = 0.014), but not elsewhere (Region III: r = -0.126, r^2^ = 0.016, p = 0.541; Region IV: r = -0.056, r^2^ = 0.003, p = 0.704).

**Fig 4:**
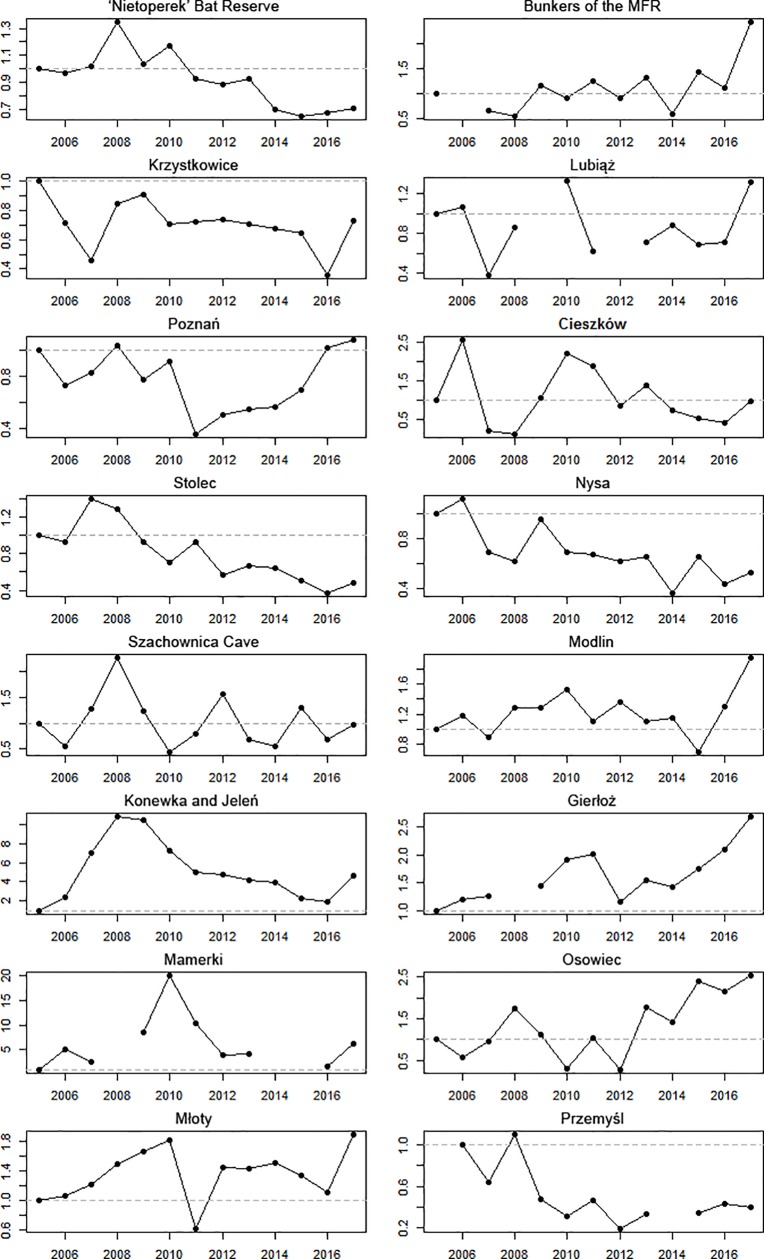
Changes in the abundance of hibernating *Barbastella barbastellus* in the hibernacula under study. Note the different scales of abundance index on the Y-axes. Horizontal, dashed lines show the abundance index for the base year; in the case of Przemyśl site it was 2006.

Analysis of changes in the numbers of individuals revealed an increase in some of the roosts, but lack of change or significant declines in others (Table 2, Fig 4). Increased numbers of individuals were recorded in roosts located in the eastern, coldest region, and in the small bunkers near MFR, with a decline apparent in the western, warmer regions and in Przemyśl (Fort I).

**Table 2:** Changes in the numbers of the barbastelles *Barbastella barbastellus* in the years 2005–2017 in the largest known hibernation sites of this species in Poland and in bunkers of the Międzyrzecz Fortification Region (MFR). Numbering of sites corresponds to that in Fig 1. Trends were evaluated using the Z value of the Mann-Kendall test (for details, see Methods). Trend symbols: ↔ no trend, ↑↑ or ↓↓—increasing or decreasing trend at significance level of 0.05, ↑ or ↓—increasing or decreasing trend at significance level of 0.1.

No.	Site	Thermal region	Study period	Median, min-max numbers of individuals	Trend	Z	p
1	“Nietoperek” Bat Reserve (main system of the MFR)	Region I	2005–2013, 2016–2017, 2014–2015*	970, 677–1409	↓↓	-2.501	0.012
2	Bunkers of the MFR(small sites)	Region I	2005–2017	88, 46–201	↑	1.787	0.074
3	“Barbastelle tunnel” near Krzystkowice	Region I	2005–2017	1331, 669–1870	↓	-1.769	0.074
4	Fort I and II in Poznań	Region II	2005–2008, 2010, 2012–2017	291, 177–379	↔	0.061	0.951
5	Monastery in Lubiąż	Region I	2005–2008, 2010–2011, 2013–2017	77, 34–118	↔	0.000	1.000
6	Ice cellar in Cieszków	Region II	2005–2017	47, 6–122	↔	-0.915	0.360
7	Adit in Stolec Rocks	Region II	2005–2017	97, 52–195	↓↓	-3.545	< 0.001
8	Nysa forts	Region II	2005–2017	96, 53–163	↓↓	-3.001	0.003
9	Szachownica cave	Region II	2005–2017	400, 184–922	↔	-0.367	0.714
10	Modlin forts	Region III	2005–2014, 2016–2017	527, 314–871	↔	0.915	0.360
11	Konewka and Jeleń	Region III	2005–2017	666, 144–1551	↔	-1.281	0.200
12	Gierłoż	Region IV	2005–2007, 2009–2017	286, 192–514	↑↑	2.674	0.007
13	Mamerki	Region IV	2005–2007, 2009–2013, 2017	84, 18–361	↔	0.358	0.721
14	Central fort of the Osowiec Fortress	Region IV	2005–2017	174, 44–397	↑↑	2.257	0.024
15	Round tunnel in Młoty	Region IV	2005–2017	292, 126–386	↔	1.159	0.246
16	Przemyśl (Fort I)**	Region III	2006–2017	105, 47–263	↓	-1.713	0.087

* Data: [72, 73].

** Data: [58]

Barbastelles in large and small roosts within the same thermal region.

The numbers of barbastelles recorded in the 47 stand-alone small bunkers (around the MFR and the main system of underground corridors of the MFR (see Methods) were not significantly correlated (Spearman rho = -0.455, p = 0.137). Indeed, an increase was found in the former site, and a significant decrease in the latter (Table 2, Fig 4).

## Discussion

The barbastelle is known for its large annual fluctuations in the number of wintering individuals [49, 57]. Analysis of long-term changes in barbastelle abundance in Poland prior to 2005 indicated that the number of barbastelles was stable, or had even increased [57]. This assessment was supported by a 30-year study of one of the largest wintering sites of barbastelles in Poland [50], and also by data from the Czech Republic [49]. Such a trend could be associated with the restoration of the population after a period when bats had been decimated due to the use of pesticides, including DDT [74]. Also, climate warming causes expansion of ranges and increases in abundance of many Lepidoptera species that make up 92% of the barbastelle diet [26, 75, 76, 77]. Increased food availability, especially in the case of pregnant and lactating females with their high energy demands, and young bats with their lack of experience in searching for and hunting of prey, may translate into an increase in the number of barbastelles. Individuals may also be better prepared for winter when more food is available (as they can accumulate fat faster).

Our results showed an overall stable trend in the number of barbastelles in Poland, but the situation varied in different thermal regions of the country. However, it should be noted that twice during the study period the total number of individuals increased dramatically year to year–by almost 3,000 between 2007 and 2008 and by almost 2,000 between 2016 and 2017 (Fig 2). Bats are slow breeders and it does not seem plausible to assume such change is just a result of reproduction–more probably the explanation is that large hibernacula are used by a variable part of the total population, depending at least partially on weather conditions–e.g., the high number of barbastelles in the 2017 survey coincided with very low temperatures in January.

### Barbastelle populations in Poland and Europe

Analysis of bat count results from hibernacula situated in different regions of Poland revealed that the number of barbastelles significantly increased in the colder east of the country (Region IV) but decreased in the warmer west (Regions I and II). In the central part of Poland (Region III) the trend could only be classified as “uncertain”. These differences were probably associated with an increase of mean winter temperatures and a decrease of the number of frost days that both varied across the country.

Mean winter temperatures recorded at the IMGW stations near barbastelle hibernacula in the years 2005–2017 did not show a significant trend across the country nor in separate thermal regions, probably due to the relatively short periods of observation, but no trend was also found in similar data from the Czech Republic [49]. However, variation in mean temperatures can influence the number of barbastelles in hibernacula. In two out of the four thermal regions of Poland (Regions I and II), weak but significant correlations were noted between the number of hibernating barbastelles and mean temperature, with the number of bats in hibernacula decreasing with increasing winter temperature. Earlier studies found increasing trends in the number of barbastelles wintering in some of the sites included also in the present study–the MFR, Poznań Fort I, Modlin forts and Szachownica cave–until 2004 [57, 50], Our data from subsequent winters indicated a declining trend in the MFR (he main system) and stable numbers at the other three sites.

The number of barbastelles seems to be decreasing in many regions in the west of Europe [51, 52, 53, 54, 55]. This decrease is generally attributed to the reduction of the area of old deciduous forests which provide shelters and foraging places. However, conclusions about population trends are based mainly on the results of bat counts in hibernacula. Our results show that climate warming may be an important reason for the decrease in the number of barbastelles in large hibernacula in the countries located to the west of Poland. It is thus possible that in some regions, where the condition of the forests has not changed, conclusions may not be drawn correctly. In the western part of Europe, mean temperatures in winter are higher and winter is shorter than in the eastern part of the continent [11, 14, 78]. Although the temperature rise in winter months progresses faster in eastern Europe than in the west, winters are still cooler there [13, 14, 15, 79, 80]. There are no many data on the number of barbastelle in Eastern Europe (Russia, Ukraine, Belarus), but published results of winter roost surveys indicate that the population is small but stable [56, 81]. The results of winter counts in the West Europe, where the barbastelle populations are declining [52, 53, 54] and from the east of the continent where it is stable [56, 81], suggest that the increase in the temperature of winter months may influence the selection of winter roosts by barbastelles. Therefore, inference about the trends in the population of the species should ideally be based not only on winter count bats, but also on observations outside the winter period e.g. searching and monitoring barbastelle maternity colonies. Although the number of barbastelles in many underground roosts of Central Europe (Poland, Germany, Czech Republic) is declining ([48], data in this paper), maternity colonies still exist or new colonies are found [82, 83, 84]. In Poland, new maternity colonies are being found also near hibernacula (e.g. the ice cellar in Cieszków, the adit in Stolec Rock), where significant decrease in the number of wintering barbastelles was found ([84], data present in the article).

### Behavioural response to climate change

Rapid changes in behaviour in response to climatic trends may be expected in a species such as the barbastelle—a cryophilic bat that does not migrate over long distances [7, 26]. The same is true for birds, in which larger changes in populations associated with climate change were reported for short-distance migrants [85]. Therefore, fewer and fewer barbastelles may be recorded in large, regularly monitored, underground hibernacula. Such a phenomenon would be mainly noted in the warmest regions of Poland (Region I and II), where a moderate decline in number of individuals was found, and to a lesser extent in the areas where the climate warming effect is currently weakest (cold regions). It is true that mean winter temperatures analysed in our study did not show significant trends, but an analysis of 50-year dataset revealed a significant increase, most pronounced in the western part of Poland [15, 86, 87]. The mean December temperatures in different regions of country were similar, but the greatest increase in temperature was observed in January and February. In western Poland, the disappearance of the thermal winter has been noted [59, 87, 88] alongside an increase in the number of barbastelles in small structures that cool more rapidly, which was indicated by our results from the bunkers of the MFR.

Barbastelles can withstand minimum temperatures of -9°C [25] and most probably begin hibernation in smaller, shallower sites, which cool more rapidly. By delaying their arrival in larger underground roosts until these become sufficiently cold, the animals can probably reduce fat consumption [89]. Previous research showed that bats declined in body mass more rapidly early in the season when ambient temperature in hibernaculum was higher. The rate of estimated fat consumption decreased as hibernation progressed and as ambient temperature declined within the hibernaculum [31, 90]. Studies of temperature fluctuations in large and small winter roosts and more detailed data on the dynamics of bat numbers present would be needed to fully understand the impact of temperature on the choice of hibernation conditions by bats. Temperatures in January may exert a remarkable effect on the number of barbastelles in large hibernacula. Frost spells, depending on their duration and/or minimum temperatures, may flush different numbers of individuals out of shallow roosts, thus affecting the results of censuses. Such a situation took place in the season of 2016/2017, when the weather in December was similar to that of the preceding year, while January was much colder and almost 2000 more individuals were noted in large wintering sites. Weather conditions in February most probably had no significant effect on the number of barbastelles hibernating in large hibernacula, as our counts were restricted to the first half of the month. However, increasing temperatures in February may contribute to a shortening bat hibernation, or at least to earlier departure of the animals from large wintering sites to some transitional roosts.

### Conservation of barbastelles

Increased climate warming, especially in winter [11, 12, 15], will most probably lead to changes in the thermal regime of traditional bat hibernacula [44] and, consequently, to bats searching for colder places to survive the winter. This may mean that fewer and fewer barbastelles are recorded in large, regularly monitored hibernacula. The method of estimating population trends of this species, based on long-term winter censuses in large underground structures, may become less reliable. Unfortunately, such censuses still seem to be the most effective (if not only) way of estimating populations of “forest” bat species which outside of the hibernation period use scattered, frequently switched and hard to find tree roosts. In Poland, the problem seems to be restricted to the warmer, western part of the country–or maybe western and central parts. This situation may change in the years to come, especially if the temperatures of February keep on increasing [15, 88].

Future research should focus include monitoring temperatures within the wintering sites and, because barbastelles rapidly respond to changes in temperature, bat censuses should ideally be preceded by several frost days [30, 91], as indicated by the 2017 data in this paper. As inclusion of smaller transitional and winter roosts (largely unknown and in many cases inaccessible for humans) into monitoring schemes does not seem possible, careful adjustment of bat count dates to weather conditions in individual thermal regions, instead of using just one period for the whole country, could make survey results more reliable in the case of barbastelles and other cryophilic bats.

Climatic changes and their consequences for species and their habitats should be considered when undertaking protective measures and monitoring population status. In the case of barbastelles, whose monitoring is mainly based on estimations of their number in wintering sites, the development of methods to monitor population in the breeding period–at least in selected foraging areas—would be very valuable.

## Supporting information

S1 Appendix Coordinates of the barbastelle hibernacula and the nearest meteorological station, mean winter temperature and number of frost days in winter (Excel). (XLS)
